# Positive predictive value of medical student specialty choices

**DOI:** 10.1186/s12909-018-1138-x

**Published:** 2018-03-09

**Authors:** M. Douglas Jones, Traci Yamashita, Randal G. Ross, Jennifer Gong

**Affiliations:** 1 Department of Pediatrics, University of Colorado School of Medicine, MS 8402, Ed 2 South, Rm L28-4311, 13121 East 17th Ave, Aurora, CO 80045 USA; 2 University of Colorado School of Medicine, Aurora, CO USA; 3 Department of Psychiatry, University of Colorado School of Medicine, Aurora, CO USA; 4 Department of Family Medicine, University of Colorado School of Medicine, Aurora, CO USA

**Keywords:** Undergraduate medical education, Medical students, Career choice, United States medical licensing examination

## Abstract

**Background:**

Although medical school programs oriented toward postgraduate specialty training have the potential to reduce the duration and cost of medical school for US medical students, success depends on the ability of students to predict their postgraduate specialties. It is clear that first-year choices are poorly predictive, but it is not known when predictions become sufficiently reliable to support specialty-oriented learning programs. We therefore examined the predictive value of specialty preferences expressed at the ends of the first, second and third years of medical school and asked whether concurrent expressions of confidence in choices improved predictive ability. We also investigated the possibility that discrepancies between predicted and actual postgraduate specialty training were related to scores on an examination of knowledge in basic biomedical sciences required for US medical school graduation (the United States Medical Licensing Examination (USLME) Step 1 examination).

**Method:**

We calculated positive and negative predictive values (PPV and NPV, respectively) for specialty choices and the sensitivity and specificity of asking for choices for 634 University of Colorado School of Medicine students who trained in 23 accredited residencies from 2011 through 2015. We examined the effect of confidence in first choices in 609 students, and in 334 students, sought an association between USMLE Step 1 scores and switching from postgraduate training specialties predicted at the end of year 2.

**Results:**

The PPV of first choices improved from years 1 through 3. NPV was high throughout. PPVs of year 3 first choices ranged from 79% in Anesthesiology to 95% in Psychiatry. Expressions of confidence in first choices did not improve PPV. Sensitivity of asking for first choices increased with time; specificity was consistently high. USLME Step 1 scores were higher for students who ultimately trained in specialties more competitive than first-choice specialties at the end of year 2.

**Conclusions:**

Specialty-oriented learning programs during medical school must accommodate students who change career plans. The PPV of specialty first choices improves each year, but even year 3 predictions can be inaccurate with potential loss of students from specialty-specific programs. USMLE Step 1 scores appeared to affect career plans expressed at the end of year 2.

**Electronic supplementary material:**

The online version of this article (10.1186/s12909-018-1138-x) contains supplementary material, which is available to authorized users.

## Background

The rationale for early educational specialization in US medical schools is compelling. Ideally, schools would be able to identify students for focused learning ending with postgraduate residency training in the chosen specialty [1, 2]. Proposed approaches include specialty-specific longitudinal medical school experiences integrated with postgraduate training in that specialty [1, 2]. The ideal outcome would be fewer years of training.

A vulnerability of specialty-specific programs is that students will enter a program and then change career plans, disruptive for both student and school. It would thus be important to know when predicted postgraduate training choices become reliable. It is clear that this is not the case in the first year [3–9]. Recent data from the Association of American Medical Colleges (AAMC) show that approximately one-quarter of specialties identified on the AAMC Graduation Questionnaire (GQ) are the same as those chosen by the same students on the Matriculating Student Questionnaire (MSQ) [10]. Fewer studies have examined the accuracy of second-year choices [3, 7, 9, 11, 12]. Data from the third year are rare. A Canadian program that offered fourth-year tracks in medicine, surgery, psychiatry and family medicine was soon abandoned because students chose postgraduate specialties different from their fourth-year tracks [13]. In contrast, a Missouri program that tracks students into family medicine during the fourth year [14] continues to be successful (Personal communication, Professor Erika Ringdahl).

One approach to gaining insight into the utility of student predictions of postgraduate specialty training is to calculate their positive and negative predictive value (PPV and NPV, respectively) [8, 15, 16]. PPV would express the percentage of students who go on to pursue postgraduate residency training in the predicted specialty; NPV would express the percentage of students who omit a specialty choice and decline to pursue training in that specialty.

We calculated the PPV and NPV of specialty choices for 23 US medical specialties at the ends of the first, second or third year at the University of Colorado School of Medicine (CUSOM). We sought to determine if PPV and NPV changes with time and the possibility that results for some specialties or groups of primary care specialties might be better than for others. In a subset of students, we assessed whether expressions of the confidence with which they made first choices improved PPV. Finally, to examine one possible reason that students change career plans, we examined the relationship between results on an examination of knowledge in the basic biomedical sciences required for graduation from US medical schools (the United States Medical Licensing Examination (USLME) Step 1 examination [17]) and discrepancies between specialties identified as first choices at the end of year 2, prior to receipt of examination scores, and the postgraduate specialty training that those students eventually pursued. Despite studies showing little correlation between Step 1 scores and later clinical performance [18, 19], training programs in the US regularly rely on Step 1 scores to screen applicants [18–20]. We hypothesized that students might change career plans based on perceptions of how their Step 1 scores would affect acceptance in training programs.

Finally, we examined the sensitivity and specificity of inquiring about career choices. It is important to note the distinction between sensitivity and specificity, on the one hand, and PPV and NPV, on the other. Sensitivity and specificity examine the utility of asking students for choices. PPV and NPV examine the utility of the choices that students make [16].

## Methods

The study population consisted of 749 students at the CUSOM for whom we could determine postgraduate residency training during the years 2011 through 2015 (Additional file 1: Figure S1). We included students matching [21] in Preliminary or Transitional Year programs [22] only if we knew their eventual training specialties and excluded Medical Scientist Training Program (MSTP) [23] students since their progression through medical school and thus their knowledge and experience with specialties differed from peers. This eliminated 45 students.

At the end of each of the first three years of medical school, we asked students to rank their first, second and third specialty choices from a list of 23 Accreditation Council of Graduate Medical Education (ACGME) [22] specialties. Not all 704 students ranked specialty choices, leaving 634 students who ranked specialty choices at least once during the three years. Positive predictive value (PPV) was defined as the number of students at the end of each year who accurately predicted their training specialty (true positives) [15, 16] divided by the total number of students predicting that specialty (true positives + false positives) multiplied by 100 to yield a percentage. Negative predictive value (NPV) was defined as the number of students at the end of each year who failed to list a residency specialty (true negatives) divided by the total number of students who failed to list the specialty (true negatives + false negatives) [15, 16] multiplied by 100. We used binomial proportions tests for independent groups to test differences in PPV, NPV, sensitivity and specificity between specialties and calculated *P* values using Fisher’s exact test (2-tailed). We restricted specialty analyses to specialties with at least 45 trainees. The top 8 training specialties from 2011 to 2015 were Internal Medicine (*n* = 140), Family Medicine (*n* = 90), Emergency Medicine (*n* = 79), Pediatrics (*n* = 74), Anesthesiology (*n* = 60), Obstetrics and Gynecology (*n* = 48), Psychiatry (*n* = 45), and General Surgery (*n* = 45). Orthopaedic Surgery, the 9th most frequently occurring specialty, had only 28 trainees over the study period, reduced to 20 after eliminating students with missing data. Therefore, due to low numbers and incomplete ranking data we reported specialty results only for the top 8.

To see if ratings of confidence added predictive value, we compared the PPV of choices ranked first with the PPV of first choice *plus* the top two ratings of confidence on a Likert scale of 1 to 5 (5, my career goal; 4, one of my top possibilities; 3, one of several possibilities; 2, not ruled out, but not near the top of the list; and 1, not considering the specialty). We did not inquire about confidence if the student questionnaire burden that particular year was judged too heavy, reducing the number of students included in that analysis to 609 (Additional file 1: Figure S1). We compared PPVs with and without confidence ratings using a weighted generalized score statistic for paired design [24, 25].

We analyzed the relationship between United States Medical License Examination (USMLE) Step 1 scores [17] and discrepancies between choices at the end of year 2 (i.e., prior to knowledge of Step 1 scores) and eventual residency specialty in the 338 students who expressed choices at the end of year 2. We compared Step 1 scores of three groups: students who trained in a less competitive specialty, those who trained in an equally competitive specialty and those who trained in a more competitive specialty than the first choice specialty identified at the end of year 2. We tested group differences using the Kruskal-Wallis and Wilcoxon Rank Sums statistics. We defined competitiveness as residency positions per U.S senior, averaged over 2011-2015, using data from the National Resident Matching Program [26]. We calculated median Step 1 scores for 334 students (Four of 338 did not list a first choice.) who identified a first specialty preference at the end of year 2. Statistical analyses used SAS version 9.4 (SAS Institute, Cary, North Carolina, USA).

The study was approved by the Colorado Multiple Institutions Review Board, our local Institutional Review Board.

## Results

We evaluated 749 students who trained in one of 23 ACGME-accredited residency programs during the years 2011 to 2015 (Additional file 1: Figure S1). We excluded 45 (See Methods), leaving 704. Of these, 634 predicted at least one of their top three choices at the ends of years 1, 2 or 3. Students training in 2011 did not have the opportunity to respond at the end of years 1 and 2; students training in 2012 did not have the opportunity to respond at the end of year 1. We analyzed specialties with at least 45 total trainees and thus analyzed specialty-specific results only for Anesthesiology, Emergency Medicine, Family Medicine, Internal Medicine, Obstetrics and Gynecology, Pediatrics, Psychiatry and General Surgery (Table 1; Additional file 1: Table S1).

**Table 1:** PPV and NPV of 1st postgraduate specialty choices at the ends of medical school years 1, 2 and 3

Postgraduate training specialty	End of Year 1 *N* = 378				End of Year 2 *N* = 338				End of Year 3 *N* = 360			
	1st choice (N)	PPV (%)	Other choice (N)	NPV (%)	1st choice (N)	PPV (%)	Other choice (N)	NPV (%)	1st choice (N)	PPV (%)	Other choice (N)	NPV (%)
Anesthesiology	10	40	368	90^a^	11	55	327	89^b^	29	79	331	98^c^
Emergency Medicine	60	33^a^	318	93^a^	49	55	289	95^b^	38	82	322	98^c^
Family Medicine	25	60^a^	353	92^a^	35	60^b^	303	94^b^	49	86	311	97
Internal Medicine	74	30^a^	304	84^a^	84	38^b^	254	87^b^	63	90	297	95^c^
Obstetrics & Gynecology	15	33	363	93^a^	15	60	323	95^b^	29	83	331	98^c^
Pediatrics	58	29^a^	320	93^a^	47	51	291	96^b^	34	88	326	99^c^
Psychiatry	4	50	374	94^a^	7	86^b^	331	95^b^	21	95	339	99^c^
Surgery	53	17^a^	325	97^a^	27	44	311	98^b^	23	87	337	99^c^

*P* values for significant differences are shown in Additional files: ^a^See Additional file 1: Table S3a, ^b^See Additional file 1: Table S3b, ^c^See Additional file 1: Table S3c

**Fig. 1:**
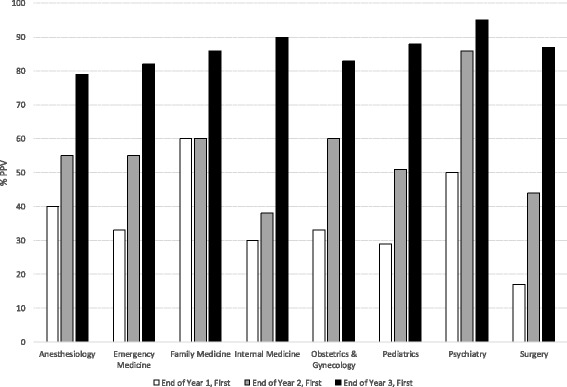
Positive predictive value (PPV) of first choice for postgraduate training specialty and by school year in which students submitted choices

PPV and NPV improved from the ends of Year 1 to 3 (Table 1; Fig. 1; Additional file 1: Table S2). We found few differences in PPV among specialties at the end of year 1, fewer at the end of year 2 and none at the end of year 3 (Table 1; Additional file 1: Tables S3a, S3b and S3c). The median PPV for year 3 was 86.5%. NPV was at or near 90% beginning with Year 1. Inter-specialty differences were significant, but small (Additional file 1: Tables S3a, S3b and S3c).

In a subset of 609 students (data not shown), we found no improvement in PPV after adding expression of confidence (see Methods), with the exception of Psychiatry at the end of Year 3, where the combination was actually *less* predictive. In that case, the difference, though statistically significant, was small (93% first choice vs 90% first choice plus expression of confidence).

We calculated the sensitivity and specificity of asking students for their choices (Table 2). Sensitivity was low until the third year. Differences between specialties (Additional file 1: Tables S3a, S3b and S3c) disappeared by the end of year 3. Specificity was high from the first year onward. We found significant, but small, differences among specialties (Additional file 1: Tables S3a, S3b and S3c).

**Table 2:** Comparison of Sensitivity and Specificity for inquiries regarding 1st specialty choices at the ends of medical school years 1, 2 and 3

Postgraduate training specialty	End of Year 1 *N* = 378				End of Year 2 *N* = 338				End of Year 3 *N* = 360			
	No. trained in specialty	% Sensitivity	No. not trained in specialty	% Specificity	No. trained in specialty	% Sensitivity	No. not trained in specialty	% Specificity	No. trained in specialty	% Sensitivity	No. not trained in specialty	% Specificity
Anesthesiology	42	10^a^	336	98^a^	41	15^b^	297	98^b^	29	79	331	98
Emergency Medicine	43	47^a^	335	88^a^	41	66^b^	297	93^b^	36	86	324	98^c^
Family Medicine	45	33^a^	333	97^a^	40	53^b^	298	95^b^	51	82	309	98^c^
Internal Medicine	72	31^a^	306	83^a^	64	50^b^	274	81^b^	71	80	289	98
Obstetrics & Gynecology	30	17^a^	348	97^a^	24	38^b^	314	98^b^	29	83	331	98
Pediatrics	38	45^a^	340	88^a^	35	69^b^	303	92^b^	34	88	326	99
Psychiatry	24	8^a^	354	99^a^	22	27^b^	316	100^b^	24	83	336	100^c^
Surgery	19	47^a^	359	88^a^	18	67^b^	320	95^b^	23	87	337	99^c^

*P* values for significant differences are shown in Additional files: ^a^See Additional file 1: Table S3a, ^b^See Additional file 1: Table S3b, ^c^See Additional file 1: Table S3c

Almost half (29 of 61) of students who trained in one of the primary care specialties (Medicine, Pediatrics and Family Medicine) had initially chosen one of the other two. We analyzed the PPV of year 2 choices of Family Medicine, Internal Medicine or Pediatrics for training in one of the three. We found PPVs of 74, 54 and 74%, respectively; NPVs were 63, 63 and 64%, respectively (Table 3). The PPV for training in one of the three primary care specialties was significantly lower for Internal Medicine than for Family Medicine or Pediatrics (*p* = 0.04 and 0.02, respectively). In each case, the PPV of training in one of the three was significantly (*p* < 0.0001) better than the PPV for training in the original year 2 choice (Table 1).

**Table 3:** Step 1 scores for students with postgraduate training in specialties less, equal or more competitive than 1st choices at end of year 2

Postgraduate Training Specialty	Positions per US Senior^26^	Specialty less competitive than year 2 choice			Specialty equally competitive as year 2 choice			Specialty more competitive than year 2 choice			Kruskal-Wallis *P*
		*N*	%	Step 1 Score, Median [IQR]^a^	*N*	%	Step 1 Score, Median [IQR]	*N*	%	Step 1 Score, Median [IQR]	
All Specialties	–	95	28.4	228 [208, 239]	150	44.9	228 [217, 243]	89	26.7	237 [218, 248]	0.02^a^
Anesthesiology	1.37	17	41.5	228 [215, 238]	6	14.6	244 [225, 245]	18	43.9	230 [221, 243]	0.29
Emergency Medicine	1.17	1	2.4	212 [n/a]	27	65.9	230 [217, 238]	13	31.7	241[220, 256]	0.11
Family Medicine	2.20	19	47.5	206 [199, 223]	21	52.5	225 [209, 236]	0	0	–	0.05^b^
Internal Medicine	1.88	28	43.8	233 [224, 246]	32	50.0	238 [222, 248]	4	6.3	245 [239, 252]	0.39
Obstetrics & Gynecology	1.22	3	13.0	228 [219, 229]	9	39.1	237 [231, 242]	11	47.8	217[207, 237]	0.16
Pediatrics	1.42	3	8.6	229 [186, 246]	24	68.6	223 [215, 236]	8	22.9	223[209, 238]	0.97
Psychiatry	1.81	9	40.9	210 [200, 230]	6	27.3	206 [205, 221]	7	31.8	219 [201, 244]	0.76
Surgery	1.12	2	11.1	230 [223, 236]	12	66.7	222 [212, 230]	4	22.2	243 [234, 248]	0.14

*IQR* Interquartile Range

^a^Wilcoxon Rank Sums test for pairwise difference between more vs less competitive groups, *P* = 0.004; more vs equally competitive groups, *P* = 0.05

^b^Wilcoxon Rank Sums test for pairwise difference between less vs equally competitive groups, *P* = 0.05

USMLE Step 1 scores for CUSOM students were virtually identical to scores for all US and Canadian graduates as reported by the National Resident Matching Program (NRMP) (Additional file 1: Table S4). After rating all 23 ACGME residency programs for competitiveness by comparing numbers of applicants to numbers of NRMP residency slots (see Methods), we compared Step 1 scores of 334 students who trained in specialties less, equally or more competitive than their year 2 first choices (Table 3; Additional file 1: Table S5). Median Step 1 scores for students who trained in residencies more competitive than Year 2 first choices were higher than scores of students who trained in residencies that were as or less competitive. Numbers of students who trained in residencies less competitive than year 2 first choices were small except for Anesthesiology, Internal Medicine and Family Medicine. Family Medicine is the least competitive specialty [26]; Step 1 scores were higher for students who identified Family Medicine at the end of year 2 than for those who trained in Family Medicine after initially choosing differently.

## Discussion

We used positive and negative predictive value to examine the ability of CUSOM students to predict postgraduate residency training. Most previous data focus on predictions by first-year students [3, 5–8, 10] with fewer data for second-year [3, 7, 9, 11, 12] and virtually none for third-year. We are to our knowledge the first to conduct a systematic examination of the ability of specialty choices at the end of year 3 to predict postgraduate residency training. We found that a noticeable proportion of students matched [21] in specialties different from those identified at the end of year 3, just a few months previously. We believe that this is the first systematic examination of the relationship of Step 1 USMLE scores [17] to discrepancies between specialties identified at the end of year 2, just prior to receipt of USLME Step 1 scores, and eventual residency training. Results are consistent with an effect of Step 1 scores on career plans. We also found differences in Step 1 scores between students who predicted training in Family Medicine at the end of year 2 and those who trained in Family Medicine after initially choosing a more competitive specialty.

We focused on PPV and NPV rather than sensitivity and specificity. Sensitivity and specificity describe the utility of the test [16], in this case the utility of asking students to predict their postgraduate training. They estimate the probability that students who pursue, or fail to pursue, postgraduate training in a particular specialty will have chosen, or failed to choose, that specialty in advance. They begin with training and look backward to see if it was predicted. PPV and NPV focus on the utility of the choices by estimating the probability that a choice for or against a specialty will be predictive. As a practical matter, schools that ask students to choose specialties have accepted the utility of asking the question. They need to know the reliability of the answer.

Although PPV is ideal for our purpose, one must be careful about generalizing PPV results. PPV and NPV change with the prevalence of the item of interest in the population [15, 16], in this case the percentage of students who pursue a particular specialty. Minor differences are not important, but it would be inappropriate to extrapolate results to schools where students are much more or less likely to pursue training in that specialty [15, 16].

Returning to whether it is useful to ask students to choose postgraduate residency training specialties, the data indicate that it is not worthwhile to ask our first- or even second-year students. Many who eventually chose the specialty were missed (low sensitivity) and choices were unreliable (low PPV). Most who did not choose the specialty were identified (high specificity) and most negative answers were reliable (high NPV). However, the latter are not especially useful to educators trying to identify candidates for focused experiences. It is not until the end of the third year that most students who trained in a specialty were identified (high sensitivity) and most who chose a specialty proceeded accordingly (high PPV).

Our results are consistent with studies suggesting that year 2 career decisions are more predictive than those expressed earlier [3, 7, 11, 12]. Nevertheless, specialty-specific learning for CUSOM year 2 students would have to anticipate substantial numbers of dropouts and provide support accordingly. The PPV of year 3 choices is better, but with PPVs varying from 79 to 95% (Table 1), up to 1 in 5 year 3 students changed training plans within the following few months, with a median estimate of 1 in 7 based on the median PPV of 86.5%.

Scott et al. analyzed factors changes in career preference during the preclinical years in Canada and found that ease of entry into a postgraduate training program was important [6]. For many US programs, despite lack of evidence that USLME scores predict clinical performance [18, 19], entry depends on USMLE Step 1 scores [18–20], especially in more competitive specialties [20]. Although one recent study found that training applicants did not seem concerned about how their USLME Step 1 scores would affect their applications, Prober et al. [18], citing personal experience, stated that students at their school seem “regularly” to change plans based on Step 1 scores. Analyzing all 334 students (including the few pursuing highly competitive specialties) who identified a first choice at the end of year 2, we found significantly higher Step 1 scores among students training in specialties that were more competitive than those identified at the end of year 2. However, when we restricted analysis to students who trained in Anesthesiology, Emergency Medicine, Family Medicine, Internal Medicine, Obstetrics and Gynecology, Pediatrics, Psychiatry and Surgery, we found no such relationship. This is consistent with the hypothesis that concern about Step 1 scores is prominent in students considering highly competitive specialties, but less concerning to students considering less competitive specialties, except perhaps for those training in Family Medicine. Students with low Step 1 scores may have switched into Family Medicine because they decided that their Step 1 scores were less suited to more competitive residencies. Both findings support suggestions that some [18], though not all [27], students change clinical career plans based on the results of an examination of knowledge in basic biomedical sciences.

We found that the PPVs of a first choice for Family Medicine and Pediatrics at the end of year 2 were moderately predictive of training in primary care, with PPVs of 74%. However, 1 in 4 eventually chose otherwise and the PPV of a year 2 choice for Internal Medicine was just 54%. Factors underlying a decision to enter primary care and the stability of early choices have been studied extensively (see, for example, Glasser et al. [4]and Compton et al. [9]).

We are aware of another study analyzing the PPV and NPV of career choices. Looney et al. [8] studied the PPV and NPV of admission choices for generalist careers or rural-based practice. They found as did we that NPV was better than PPV. Students seem to be better at predicting what they will not choose than what they will.

This study’s limitations include, first, that PPVs for second- and third-year students might have been higher if we offered attractive career-specific tracks or inducements such as tuition forgiveness or early graduation [1, 2, 14] and higher if we had been able to include other student characteristics [8, 12, 28, 29]. Second, even though the number of students in this study is large for US studies of this type, numbers in individual specialties were small, precluding analyses in specialties chosen by few students. Although the relationship between Step 1 scores and changes in year 2 choices for all students (Table 3) seems secure, statistical power to detect differences in individual specialties is insufficient.

## Conclusion

This study confirms that year 2 medical student specialty choices are poorly predictive of postgraduate training specialties and adds information regarding predictions at the end of year 3. We found no effect of level of confidence associated with predictions. PPV and sensitivity were low until the end of year 3. Thus, prior to the end of year 3, many students who eventually trained in a particular specialty were missed and many did not pursue the specialty they predicted. This information may be helpful in estimating the time in medical school to offer specialty-specific learning programs. At the same time, failure to offer such programs may disappoint students whose predictions are accurate. In contrast to PPV, NPV is high from the first year on; failure to choose a specialty was highly predictive of failure to enter training in that specialty. We found evidence that scores on an examination of knowledge in basic biomedical sciences altered postgraduate training plans.

## Additional file

Additional file 1: **Table S1.** Population characteristics. **Table S2.** Time-related trends for Positive and Negative Predictive Value (PPV and NPV, respectively) and Sensitivity and Specificity within specialties. **Table S3a.** End of Year 1 – *P* values for differences in Positive Predictive value (PPV), Negative Predictive Value (NPV), Sensitivity (Sens) and Specificity (Spec) of 1st choice among specialties. **Table S3b.** End of Year 2 – P values for differences in Positive Predictive value (PPV), Negative Predictive Value (NPV), Sensitivity (Sens) and Specificity (Spec) of 1st choice among specialties. **Table S3c.** End of Year 3 – P values for differences in Positive Predictive value (PPV), Negative Predictive Value (NPV), Sensitivity (Sens) and Specificity (Spec) of 1st choice among specialties. **Table S4.** Step 1 scores by match specialty for 2014-15 First Year Residents who graduated from U.S. and Canadian medical schools and for University of Colorado School of Medicine (CUSOM) students who matched during 2011-2015. **Table S5.** Year 2 predictions (by 1st choice) of postgraduate training specialty categorized by actual postgraduate specialty. **Figure S1.** Study population flow diagram. (DOCX 102 kb)

## Abbreviations

AAMC: Association of American Medical Colleges
ACGME: Accreditation Council for Graduate Medical Education
CUSOM: University of Colorado School of Medicine
GQ: Graduation Questionnaire
MSQ: Matriculating Student Questionnaire
NPV: Negative Predictive Value
NRMP: National Resident Matching Program
PPV: Positive Predictive Value
US: United States
USMLE: United States Medical Licensing Examination
