# Supplementary material for: Positive predictive value of medical student specialty choices
Source: BMC Med Educ. 2018 Mar 9;18:33. doi: 10.1186/s12909-018-1138-x (PMC5845137; doi:10.1186/s12909-018-1138-x)
Supplement: Supplementary file 1 — Table S1. Population characteristics. Table S2. Time-related trends for Positive and Negative Predictive Value (PPV and NPV, respectively) and Sensitivity and Specificity within specialties. Table S3a. End of Year 1 – P values for differences in Positive Predictive value (PPV), Negative Predictive Value (NPV), Sensitivity (Sens) and Specificity (Spec) of 1st choice among specialties. Table S3b. End of Year 2 – P values for differences in Positive Predictive value (PPV), Negative Predictive Value (NPV), Sensitivity (Sens) and Specificity (Spec) of 1st choice among specialties. Table S3c. End of Year 3 – P values for differences in Positive Predictive value (PPV), Negative Predictive Value (NPV), Sensitivity (Sens) and Specificity (Spec) of 1st choice among specialties. Table S4. Step 1 scores by match specialty for 2014-15 First Year Residents who graduated from U.S. and Canadian medical schools and for University of Colorado School of Medicine (CUSOM) students who matched during 2011-2015. Table S5. Year 2 predictions (by 1st choice) of postgraduate training specialty categorized by actual postgraduate specialty. Figure S1. Study population flow diagram. (DOCX 102 kb) [file 12909_2018_1138_MOESM1_ESM.docx]

Additional file

Supplemental Digital Content

Table S1. Population characteristics.

| Characteristic | Responded to End of Year 1 Questionnaire | Responded to End of Year 2 Questionnaire | Responded to End of Year 3 Questionnaire | Total number of students who responded at one or more time points |
| --- | --- | --- | --- | --- |
| Number (n) | 378 | 338 | 360 | 634 |
| Postgraduate Training Year, n (%)  2011  2012  2013  2014  2015 | 0  0  117 (31.0)  125 (33.1)  136 (36.0) | 0  102 (30.2)  109 (32.3)  11 (3.3)  116 (34.3) | 110 (30.6)  13 (3.6)  1 (0.3)  110 (30.6)  126 (35.0) | 110 (17.4)  115 (18.1)  144 (22.7)  127 (20.0)  138 (21.8) |
| Postgraduate Training Specialty, n (%)  Anesthesiology  Emergency Medicine  Family Medicine  Internal Medicine  Obstetrics & Gynecology  Pediatrics  Psychiatry  Surgery-General | 42 (11.1)  43 (11.4)  45 (11.9)  72 (19.1)  30 (7.9)  38 (10.1)  24 (6.4)  19 (5.0) | 41 (12.1)  41 (12.1)  40 (11.8)  64 (18.9)  24 (7.1)  35 (10.4)  22 (6.5)  18 (5.3) | 29 (8.1)  36 (10.0)  51 (14.2)  71 (19.7)  29 (8.1)  34 (9.4)  24 (6.7)  23 (6.4) | 59 (9.3)  75 (11.8)  85 (13.4)  124 (19.6)  46 (7.3)  67 (10.6)  39 (6.2)  37 (5.8) |
| Responded to Strength of Specialty Choice item, n (%)  Anesthesiology  Emergency Medicine  Family Medicine  Internal Medicine  Obstetrics & Gynecology  Pediatrics  Psychiatry  Surgery | 226 (59.8)  238 (63.0)  232 (61.4)  245 (64.8)  227 (60.1)  236 (62.4)  225 (59.5)  235 (62.2) | 292 (86.4)  295 (87.3)  293 (86.7)  313 (92.6)  294 (87.0)  300 (88.8)  296 (87.6)  301 (89.1) | 185 (51.4)  190 (52.8)  183 (50.8)  199 (55.3)  186 (51.7)  188 (52.2)  190 (52.8)  185 (51.4) | 547 (86.3)  556 (87.7)  547 (86.3)  582 (91.8)  543 (85.7)  560 (88.3)  547 (86.3)  561 (88.5) |

Table S2. Time-related trends for Positive and Negative Predictive Value (PPV and NPV, respectively) and Sensitivity and Specificity within specialties (Cochran-Mantel-Haenszel test with time as ordinal).

| Postgraduate Training Specialty | End of Year 1 | | End of Year 2 | | End of Year 3 | | P value | |
| --- | --- | --- | --- | --- | --- | --- | --- | --- |
|  | PPV (%) | NPV (%) | PPV (%) | NPV (%) | PPV (%) | NPV (%) | PPV | NPV |
| Anesthesiology | 40 | 90 | 55 | 89 | 79 | 98 | .02 | <.0001 |
| Emergency Med | 33 | 93 | 55 | 95 | 82 | 98 | <.0001 | .0005 |
| Family Medicine | 60 | 92 | 60 | 94 | 86 | 97 | .009 | .003 |
| Internal Medicine | 30 | 84 | 38 | 87 | 90 | 95 | <.0001 | <.0001 |
| Obstetrics & Gynecology | 33 | 93 | 60 | 95 | 83 | 98 | .001 | .0006 |
| Pediatrics | 29 | 93 | 51 | 96 | 88 | 99 | <.0001 | .0004 |
| Psychiatry | 50 | 94 | 86 | 95 | 95 | 99 | .02 | .002 |
| Surgery | 17 | 97 | 44 | 98 | 87 | 99 | <.0001 | .04 |

| Postgraduate Training Specialty | End of Year 1 | | End of Year 2 | | End of Year 3 | | P value | |
| --- | --- | --- | --- | --- | --- | --- | --- | --- |
|  | Sensitivity | Specificity | Sensitivity | Specificity | Sensitivity | Specificity | Sensitivity | Specificity |
| Anesthesiology | 10 | 98 | 15 | 98 | 79 | 98 | <.0001 | .98 |
| Emergency Medicine | 47 | 88 | 66 | 93 | 86 | 98 | .0003 | <.0001 |
| Family Medicine | 33 | 97 | 53 | 95 | 82 | 98 | <.0001 | .63 |
| Internal Medicine | 31 | 83 | 50 | 81 | 80 | 98 | <.0001 | <.0001 |
| Obstetrics & Gynecology | 17 | 97 | 38 | 98 | 83 | 98 | <.0001 | .22 |
| Pediatrics | 45 | 88 | 69 | 92 | 88 | 99 | .0001 | <.0001 |
| Psychiatry | 8 | 99 | 27 | 100 | 83 | 100 | <.0001 | .57 |
| Surgery | 47 | 88 | 67 | 95 | 87 | 99 | .006 | <.0001 |

Table S3a. End of Year 1 – P values for differences in Positive Predictive value (PPV), Negative Predictive Value (NPV), Sensitivity (Sens) and Specificity (Spec) of 1^st^ choice among specialties. (ANES – Anesthesiology; EM – Emergency Medicine; FM – Family Medicine; IM – Internal Medicine; OBGYN – Obstetrics & Gynecology; PEDS – Pediatrics; PSYCH – Psychiatry; SURG – Surgery)

|  | ANES | EM | FM | IM | OBGYN | PEDS | PSYCH | SURG |
| --- | --- | --- | --- | --- | --- | --- | --- | --- |
| ANES PPV  NPV  Sens  Spec |  | .73  .18  **.0002**  **<.0001** | .45  .45  **.009**  .32 | .49  **.02**  **.01**  **<.0001** | 1.00  .11  .48  .45 | .49  .10  **.0007**  **<.0001** | 1.00  **.03**  1.00  .17 | .19  **<.0001**  **.002**  **<.0001** |
| EM PPV  NPV  Sens  Spec |  |  | **.03**  .57  .28  **<.0001** | .71  **.0004**  .11  .07 | 1.00  .88  **.01**  **<.0001** | .69  .76  1.00  1.00 | .60  .54  **.001**  **<.0001** | .05  **.02**  1.00  .91 |
| FM PPV  NPV  Sens  Spec |  |  |  | **.009**  **.003**  .84  **<.0001** | .19  .48  .18  **.02** | **.01**  .38  .37  **<.0001** | 1.00  .20  **.04**  **.02** | **.0002**  **.003**  .40  **<.0001** |
| IM PPV  NPV  Sens  Spec |  |  |  |  | .77  **.0001**  .22  **<.0001** | 1.00  **.0001**  .15  .09 | .58  **<.0001**  **.03**  **<.0001** | .14  **<.0001**  .18  .10 |
| OBGYN PPV  NPV  Sens  Spec |  |  |  |  |  | .76  .88  **.02**  **<.0001** | .60  .65  .44  **.02** | .28  **.02**  **.03**  **<.0001** |
| PEDS PPV  NPV  Sens  Spec |  |  |  |  |  |  | .58  .75  **.004**  **<.0001** | .18  **.04**  1.00  1.00 |
| PSYCH PPV  NPV  Sens  Spec |  |  |  |  |  |  |  | .16  .10  **.005**  **<.0001** |

Table S3b. End of Year 2 – P values for differences in Positive Predictive value (PPV), Negative Predictive Value (NPV), Sensitivity (Sens) and Specificity (Spec) of 1^st^ choice among specialties. (ANES – Anesthesiology; EM – Emergency Medicine; FM – Family Medicine; IM – Internal Medicine; OBGYN – Obstetrics & Gynecology; PEDS – Pediatrics; PSYCH – Psychiatry; SURG – Surgery)

|  | ANES | EM | FM | IM | OBGYN | PEDS | PSYCH | SURG |
| --- | --- | --- | --- | --- | --- | --- | --- | --- |
| ANES PPV  NPV  Sens  Spec |  | 1.00  **.007**  **<.0001**  **.001** | 1.00  .06  **.0004**  **.04** | .34  .51  **.0003**  **<.0001** | 1.00  **.005**  .06  1.00 | 1.00  **.001**  **<.0001**  **.0007** | .32  **.005**  .31  .11 | .72  **<.0001**  **.0001**  **.04** |
| EM PPV  NPV  Sens  Spec |  |  | .82  .48  .26  .17 | .07  **.002**  .16  **<.0001** | .78  1.00  **.04**  **.002** | .84  .55  1.00  1.00 | .22  1.00  **.004**  **<.0001** | .47  .07  1.00  .18 |
| FM PPV  NPV  Sens  Spec |  |  |  | **.04**  **.01**  .84  **<.0001** | 1.00  .38  .31  .07 | .50  .19  .24  .17 | .39  .49  .07  **.0003** | .31  **.007**  .40  1.00 |
| IM PPV  NPV  Sens  Spec |  |  |  |  | .16  **.0006**  .34  **<.0001** | .20  **.0002**  .09  **<.0001** | **.02**  **.0008**  .08  **<.0001** | .65  **<.0001**  .29  **<.0001** |
| OBGYN PPV  NPV  Sens  Spec |  |  |  |  |  | .57  .69  **.03**  **.0009** | .35  1.00  .54  .07 | .52  .07  .12  .07 |
| PEDS PPV  NPV  Sens  Spec |  |  |  |  |  |  | .12  .56  **.003**  **<.0001** | .63  .22  1.00  .14 |
| PSYCH PPV  NPV  Sens  Spec |  |  |  |  |  |  |  | .09  .05  **.02**  **.0004** |

At end of year 2: Primary Care group PPV = 64 vs Primary Care IM PPV = 54, p=.13; vs Primary Care FM PPV=74, p=.33; vs Primary Care Peds PPV=54, p=.22

At end of year 2: Primary Care group NPV = 81 vs Primary Care IM NPV = 63, p<.0001; vs Primary Care FM NPV=63, p<.0001; vs Primary Care Peds NPV=64, p=.0001Table S3c. End of Year 3 – P values for differences in Positive Predictive value (PPV), Negative Predictive Value (NPV), Sensitivity (Sens) and Specificity (Spec) of 1^st^ choice among specialties. (ANES – Anesthesiology; EM – Emergency Medicine; FM – Family Medicine; IM – Internal Medicine; OBGYN – Obstetrics & Gynecology; PEDS – Pediatrics; PSYCH – Psychiatry; SURG – Surgery)

|  | ANES | EM | FM | IM | OBGYN | PEDS | PSYCH | SURG |
| --- | --- | --- | --- | --- | --- | --- | --- | --- |
| ANES PPV  NPV  Sens  Spec |  | 1.00  1.00  .52  .79 | .54  .44  .77  .78 | .18  **.04**  1.00  1.00 | 1.00  1.00  1.00  1.00 | .49  .75  .49  .75 | .14  .54  1.00  .07 | .71  .34  .71  .07 |
| EM PPV  NPV  Sens  Spec |  |  | .77  .29  .77  1.00 | .23  **.03**  .60  1.00 | 1.00  1.00  .74  .57 | .52  .75  1.00  .38 | .10  .75  1.00  **.03** | .73  .50  1.00  **.03** |
| FM PPV  NPV  Sens  Spec |  |  |  | .55  .29  .82  1.00 | .75  .28  1.00  .57 | 1.00  .17  .55  .37 | .17  .16  1.00  **.03** | 1.00  .08  .74  **.03** |
| IM PPV  NPV  Sens  Spec |  |  |  |  | .31  **.03**  1.00  .76 | .74  **.01**  .41  .53 | .45  **.008**  1.00  .05 | .70  **.005**  .55  .05 |
| OBGYN PPV  NPV  Sens  Spec |  |  |  |  |  | .72  1.00  .72  1.00 | .24  .75  1.00  .12 | 1.00  .50  1.00  .12 |
| PED PPV  NPV  Sens  Spec |  |  |  |  |  |  | .65  1.00  .71  .21 | 1.00  .72  1.00  .21 |
| PSYCH PPV  NPV  Sens  Spec |  |  |  |  |  |  |  | .22  1.00  1.00  .62 |

Table S4. Step 1 scores by match specialty for 2014-15 First Year Residents who graduated from U.S. and Canadian medical schools and for University of Colorado School of Medicine (CUSOM) students who matched during 2011-2015.

| Match Specialty | 2014-2015 First Year Residents who graduated from U.S. and Canadian medical schools | CUSOM, Matched during 2011-2015 |
| --- | --- | --- |
| Anesthesiology (N)  Step 1 Score, Median [IQR]^*^  STEP 1 Score, Mean (SD)^**^ | 769  229 [219, 241]  229.9 (15.9) | 59  229 [221, 243]  230.6 (15.8) |
| Emergency Medicine (N)  Step 1 Score3, Median [IQR]  Step 1 Score, Mean (SD) | 1439  230 [218, 242]  229.6 (16.8) | 75  227 [211, 242]  227.1 (21.1) |
| Family Medicine (N)  Step 1 Score, Median [IQR]  Step 1 Score, Mean (SD) | 1597  215 [202, 229]  216.6 (17.7) | 85  213 [204, 232]  216.7 (21.3) |
| Internal Medicine (categorical) (N)  Step 1 Score, Median [IQR]  Step 1 Score, Mean (SD) | 3509  231 [217, 245]  230.2 (18.6) | 124  236 [221, 246]  232.0 (19.3) |
| Obstetrics & Gynecology (N)  Step 1 Score, Median [IQR]  Step 1 Score, Mean (SD) | 1013  225 [213, 238]  225.2 (16.9) | 46  230 [213, 237]  225.6 (17.4) |
| Pediatrics (N)  Step 1 Score, Median [IQR]  Step 1 Score, Mean (SD) | 1983  226 [212, 238]  225.3 (17.9) | 67  226 [212, 239]  224.8 (18.7) |
| Psychiatry (N)  Step 1 Score, Median [IQR]  Step 1 Score, Mean (SD) | 743  219 [204, 234]  219.3 (18.2) | 39  214 [202, 230]  214.3 (19.3) |
| Surgery (categorical) (N)  Step 1 Score, Median [IQR]  Step 1 Score, Mean (SD) | 1010  232 [220, 244]  232 (16.2) | 37  227 [216, 239]  228.4 (18.6) |

N, number; ^*^IQR, Interquartile Range;^**^SD, Standard Deviation

Table S5. Year 2 predictions (by 1^st^ choice) of postgraduate training specialty categorized by actual postgraduate specialty.

| Actual Match Specialty | Year 2 predicted specialty less competitive than actual specialty | | | Year 2 predicted specialty more competitive than actual specialty | | |
| --- | --- | --- | --- | --- | --- | --- |
|  | 1^st^ Specialty Choice | N | % | 1^st^ Specialty Choice | N | % |
| Anesthesiology | Surgery  Plastic Surgery  Orthopaedic Surgery  Emergency Med  Ob/Gyn  Thoracic Surgery  Urology | 5  3  3  2  2  1  1 | 29.4  17.7  17.7  11.8  11.8  5.9  5.9 | Internal Medicine  Neurology  Family Med  Pediatrics  Radiology-Diagnostic | 11  3  2  1  1 | 61.1  16.7  11.1  5.6  5.6 |
| Emergency Medicine | Otolaryngology | 1 | 100.0 | Internal Med  Family Med  Pediatrics  Anesthesiology | 5  4  3  1 | 38.5  30.8  23.1  7.7 |
| Family Medicine | Internal Med  Emergency Med  Pediatrics  Surgery  Neurology  Orthopaedic Surgery  Psychiatry | 6  4  4  2  1  1  1 | 31.6  21.1  21.1  10.5  5.3  5.3  5.3 | -- | -- | -- |
| Internal Medicine | Pediatrics  Emergency Med  Anesthesiology  Ob/Gyn  Otolaryngology  Neurology  Ophthalmology  Dermatology  PM&R  Surgery | 7  5  3  3  3  2  2  1  1  1 | 25.0  17.9  10.7  10.7  10.7  7.1  7.1  3.6  3.6  3.6 | Family Med | 4 | 100.0 |
| Obstetrics & Gynecology | Emergency Med  Otolaryngology  Urology | 1  1  1 | 33.3  33.3  33.3 | Internal Med  Family Med  Pediatrics  Neurology | 5  3  2  1 | 45.5  27.3  18.2  9.1 |
| Pediatrics | Emergency Med  Orthopaedic Surgery  Surgery | 1  1  1 | 33.3  33.3  33.3 | Internal Med  Family Med | 7  1 | 87.5  12.5 |
| Psychiatry | Emergency Med  Neurological Surgery  Ob/Gyn  Pediatrics  PM&R  Radiology-Diagnostic  Surgery  Urology | 2  1  1  1  1  1  1  1 | 22.2  11.1  11.1  11.1  11.1  11.1  11.1  11.1 | Internal Med | 7 | 100.0 |
| Surgery | Neurological Surgery  Thoracic Surgery | 1  1 | 50.0  50.0 | Pediatrics  Emergency Med | 3  1 | 75.0  25.0 |

Figure S1. Study population flow diagram.

CUSOM Residency Matches

2011-2015

N = 749

End of Year 1

Numbers displayed to the right of shaded boxes indicate the total number of unique students responding to requests for specialty ranking and indication of confidence, respectively.

Rated Confidence in Decision

N = 231

Not Asked for Degree of Confidence

N = 112

Rated Confidence in Decision

N = 323

Not Asked for Degree of Confidence

N = 2

Rated Confidence in Decision

N = 257

MSTP Students

N = 20

Preliminary or Transitional Year without Subsequent Information

N = 25

N = 724

End of Year 2

N = 704

N = 634

Ranked Specialty

N = 360

Did Not Respond

N = 65

Did Not Respond

N = 17

Not Asked for Degree of Confidence

N = 113

Did Not Respond

N = 8

Ranked Specialty

N = 338

Did Not Respond

N = 108

Not Asked

N = 258

Not asked

N = 271

Did Not Respond

N = 55

Did Not Respond

N = 13

Ranked Specialty

N = 378

End of Year 3

Not Asked

N = 279

N = 609
